# Transcriptome analysis unveils the functional effects of ectomycorrhizal fungal colonization on cadmium tolerance of willow saplings

**DOI:** 10.3389/fmicb.2025.1570200

**Published:** 2025-03-18

**Authors:** Lijiao Wang, Baoshan Yang, Hui Wang, Jiaxing Shi, Jinhao Dong, Xiaoxia Zhao, Guanghua Qin, Xinhua He, Meiyuan Wang

**Affiliations:** ^1^School of Water Conservancy and Environment, University of Jinan, Jinan, China; ^2^Shandong Provincial Engineering Technology Research Center for Ecological Carbon Sink and Capture Utilization, Jinan, China; ^3^Jinan Environmental Research Academy, Jinan, China; ^4^Shandong Academy of Forestry, Jinan, China; ^5^Department of Land, Air and Water Resources, University of California at Davis, Davis, CA, United States

**Keywords:** physiochemical responses, antioxidant defense, differentially expressed genes, secondary metabolites, willow sapling

## Abstract

**Introduction:**

Ectomycorrhizal fungus (ECMF) could enhance plant tolerance to heavy metal toxicity by altering metal accumulation and protecting plants from oxidative injury. However, the molecular mechanisms underlying ECMF-mediated detoxification of cadmium (Cd) in willow sapling are not well known. This study aimed to unveil the roles of *Cenococcum geophilum (CG)* and Suillus luteus (SL) in regulating Cd toxicity tolerance in willow (*Salix psammophila* ‘Huangpi1’) saplings.

**Methods:**

This study systematically evaluated physiological and biochemical parameters in the leaf and root tissues of 18 willow saplings, while concurrently conducting transcriptomic analysis of the roots under Cd stress. The specific treatments were labeled as follows: NF (no ECMF inoculation and no Cd addition), CG (CG colonization only), SL (SL colonization only), NF+Cd (no ECMF inoculation with 100 μM Cd addition), CG+Cd (CG colonization with 100 μM Cd addition), and SL+Cd (SL colonization with 100 μM Cd addition).

**Results:**

The results showed the growth, photosynthesis, antioxidant system and transcriptome of 2-month-old willow saplings responded differently to ECMFs colonization under Cd stress. S. luteus markedly increased the aerial parts biomass, while *C. geophilum* significantly enhanced the root property indices of willow saplings under Cd stress. The highest number of differentially expressed genes (DEGs) was observed in the comparison between CG+Cd (CG colonization with 100 μM Cd addition) and NF+Cd (no ECMF inoculation with 100 μM Cd addition). *C. geophilum* colonization activated plant hormone signal transduction and carbohydrate metabolism pathways, while S. luteus enhanced the synthesis of secondary metabolites.

**Discussion:**

This study provides a molecular perspective on the mechanism of interaction between ECMFs and willow saplings under Cd stress and supports the application of ECMFs for phytoremediation of Cd-contaminated soil.

## 1 Introduction

Heavy metal (HM) pollution is a global environmental issue, mainly caused by human activities such as mining, metallurgy, fertilization, and the abuse of pesticides, posing a significant threat to the ecological environment (Xin et al., 2023). Cadmium (Cd) is one of the most prevalent HMs, easily absorbed by plants due to its high mobility and bioavailability (Yu et al., 2023). Elevated concentration of soil Cd^2+^ can inhibit plant growth and development by disrupting plant physiology process (Liu et al., 2023a). Furthermore, Cd^2+^ can reach harmful levels in human diets and accumulate in the body through the food chain, leading to various diseases such as liver damage, cancers, and kidney and itai-itai diseases (Yang et al., 2022). Consequently, the adverse effect of Cd pollution has raised widespread concern throughout the world. Cd accumulation in plants can damage genomic DNA, reduce chlorophyll content, and decrease stomatal conductance, thereby inhibiting photosynthesis (Jing et al., 2024). In addition, Cd stress generates excessive reactive oxygen species (ROS), disrupting intracellular redox balance and activating the synthesis of both enzymes and non-enzymatic antioxidants in plants (Liu et al., 2023a). Cd accumulation also negatively impacts various plant physiological processes, including CO_2_ fixation, transpiration, and secondary metabolism (Sana et al., 2024). Thus, recent studies have focused on identifying plant that can accumulate Cd and exhibit strong resistance to Cd toxicity for the use in remediating Cd-contaminated environment (Khan et al., 2017).

Compared to chemical and physical remediation technologies, phytoremediation is environmentally friendly and non-destructive for removing HMs from soil (Yu et al., 2023). However, the effectiveness of this technique greatly depends on the accessibility of plant species that can tolerate, adsorb, stabilize, and accumulate HMs, such as accumulators and hyperaccumulators (Zulfiqar et al., 2022). Although approximate 450 angiosperm species have been identified as hyperaccumulators, most are herbaceous plants that generally produce low biomass (Haider et al., 2021). Therefore, vascular woody plants have garnered more attention in phytoremediation due to their rapidly growth and higher biomass production. Studies have confirmed that plants have evolved intricate mechanisms to tolerate HMs, including physiological, biochemical, and molecular responses (Nawaz et al., 2023). However, the tolerance threshold of plants is limited. And their metabolic processes, such as gas exchange, mineral adsorption, and even DNA synthesis, can be disrupted (Jing et al., 2024). Thus, to effectively remediate HMs-polluted environments, it is essential to explore rational strategies to enhance plant tolerance to HMs.

Ectomycorrhiza fungi (ECMF) can increase the biomass production of symbiotic plants and enhance plant tolerance to various HMs (Yu et al., 2020). In mycorrhizal symbioses, plants provide mycorrhizal fungi with carbohydrates, and in turn, ECMF supply the host plant with more mineral nutrients and water, thereby increasing tolerance to various biotic or abiotic stresses (Shi et al., 2019). There are several key mechanisms by which ECMF improve plant tolerance to HMs. Firstly, ECMF can promote plant growth, increasing biomass and thereby diluting HMs concentrations (Shi et al., 2019). Secondly, ECMF mycelia can absorb and immobilize HMs in fungal cells (Balestrini and Lumini, 2018). Furthermore, ECMF can reduce HM toxicity through compartmentalization, chelation, and sequestration of HMs within plant tissues (Chot and Reddy, 2022). Although numerous studies have explored the physio-biochemical responses of plants colonized by ECMF in HM-contaminated environment, results regarding plant tolerance to HMs have been inconsistent across different ECMFs (Colpaert et al., 2011; Luo et al., 2014). Recently, combined analyses using omics technologies and physiochemical determination have revealed that Cd exposure triggers transcriptional reprogramming of genes, disrupting the function proteins and altering genes expression related to HM transportation (Xie et al., 2023). However, to our knowledge, limited transcription information is available on the mechanisms underlying the changes in Cd accumulation and plant tolerance capacity following ECMFs colonization.

Willows (*Salix* spp.) are pioneer trees with multiple ecological functions in revegetation and phytoremediation in metal contaminated environment (Han et al., 2020). Additionally, as a mycorrhizal tress species, willows form the association with various ECMFs (Baum et al., 2006). In the current study, an integrated analysis of physiology and transcriptomics was performed to test the hypothesis that critical genes involved in Cd uptake and detoxification in willow saplings are differently inducted by the association with two ECMF species. The main objectives of this study were to elucidate: (1) the physio-biochemical responses of willow saplings under Cd stress; (2) the change in key genes and metabolic pathways associated with plant tolerance through transcriptome analysis; and (3) the molecular mechanisms underlying Cd tolerance and detoxification.

## 2 Materials and methods

### 2.1 Preparation of plants and ectomycorrhizal fungi

The morphologically uniform cuttings of *Salix psammophila* ‘Huangpi1’ (cultivation code A94), each 20 cm in length, were obtained from the Shandong Academy of Forestry in Jinan, China. The cuttings were disinfected using cotton balls soaked in 75% alcohol, followed by rinsing them with distilled water several times. Subsequently, they were incubated in 2 L plastic pots with distilled water for 3 weeks to encourage rooting. After pre-treatment, the rooted cuttings were transferred to 4 L 25% Hoagland’s solution and cultivated at room temperature (25–30°C) under natural light for 3 weeks. Subsequently, the sand and soil mixture (1:1, v/v) was passed through a 2 mm sieve, sterilized in an autoclave (pressure 0.05 MPa, 121°C) for 2 h, and then loaded into plastic pots (20 × 18 × 15 cm). Each uniform rooted sapling was transplanted into pots and cultivated under natural light at 25–30°C for 2 weeks in the greenhouse of University of Jinan, with relative air humidity of 60–70%, before the inoculation with ECMFs. A total of 18 willow saplings were selected for the pot experiment. The soil used is classified as fluvo-aquic soil (FAO Soil Classification System) and contained 1.56% organic matter, 2.99 mg kg^−1^ NO_3_^−^ - N, and 2.77 mg kg^−1^ NH_4_^+^ − N.

*Suillus luteus* (*S. luteus*) and *Cenococcum geophilum* (*C. geophilum*), donated by the Inner Mongolia Agricultural University, were cultivated on a Modified Melin-Norkan’s (MMN) solid medium and subsequently in a MMN liquid culture medium, as described by Ma et al. (2014). Mycelia of *S. luteus* and *C. geophilum* grown at 24°C for over 20 days in the liquid culture, were used for fungal inoculation. Specific inoculation procedure was showed in Supplementary Text S1.

### 2.2 Experimental design and pot experiment

A total of 18 2-month-old willow saplings were divided into three groups, with each group consisting of 6 saplings inoculated with *C. geophilum* (CG), *S. luteus* (SL) or no-ECMF inoculation (Control). After achieving over 60% root colonization (Supporting Information Text S1), three saplings from each group were individually supplemented once a week with 25% Hoagland’s solution containing 100 μM CdCl_2_·5/2H_2_O. The remaining three saplings in each group received only the same Hoagland’s solution. The treatments were labeled as follows: NF (no ECMF inoculation and no Cd addition), CG (CG colonization only), SL (SL colonization only), NF + Cd (no ECMF inoculation with 100 μM Cd addition), CG + Cd (CG colonization with 100 μM Cd addition), and SL + Cd (SL colonization with 100 μM Cd addition). The saplings were cultivated in a greenhouse under the similar growth conditions as previously described. After 5 days of treatment, roots from three saplings in each group exposed to 100 μM CdCl_2_·5/2H_2_O stress were collected and stored under −20°C for RNA extraction. The remaining saplings were collected after 30 days, separated into leaves, stems, and roots washed with deionized water, and stored in a refrigerator at 4°C for subsequent analysis within 2 weeks.

### 2.3 Measurement of growth, photosynthesis parameters and chlorophyll contents

The fresh leaves, stems and roots were collected, dried at 115°C for 30 min, and then maintained at 80°C until reaching a constant weight. The root indices of sapling were analyzed using a plant root scanner (WinRHIZO 2009, Canada). Chlorophyll was extracted from fresh leaves and quantified according to Cao et al. (2007). Before harvesting the sapling, leaf photosynthesis parameters, including net CO_2_ assimilation rate (*A*), stomatal conductance (g_s_), intercellular CO_2_ molar fraction (C_i_), and transpiration rate (Tr), were determined on the third or fourth leaves from the top of saplings between 9:00 am and 12:00 am using a LC pro-SD portable photosynthesis system (LC pro-SD, ADC, Hoddesdon, United Kingdom).

### 2.4 Determination of tolerance parameters and oxidative damage estimation

Leaf and root samples (0.1 g of fresh weight) were homogenized separately in phosphate buffer (pH 7.8). The homogenate was centrifuged at 4°C, 8000 rpm for 30 min. The supernatant was stored at 4°C for the subsequent determination of physiological parameters.

Catalase activity was measured according to the method described by Aebi (1984). The reaction mixture consisted of 0.4 mL of the supernatant mixed with 3 mL phosphate buffer (pH 7.8), 2 mL distilled water and 0.6 mL of 0.1 M H_2_O_2_. The blank control contained 4 mL phosphate buffer (pH 7.8) and 2 mL distilled water to account for any non-enzyme-related changes in absorbance. The decrease in absorbance due to the decomposition of H_2_O_2_ was monitored spectrophotometrically at 240 nm, recording the absorbance every 30 s over a 5-min period.

Superoxide dismutase (SOD) activity was assessed using the pyrogallol autoxidation method, as described by Chen and Wang (2002). The reaction mixture was prepared by adding 0.01 mL of the supernatant to 2.98 mL of Tris–HCl buffer, followed by 0.01 mL of 0.05 mM pyrogallol solution. The blank control involved the reaction mixture with 2.98 mL of Tris–HCl buffer and 0.02 mL of 10 mM HCl. The absorbance was measured spectrophotometrically at 325 nm. The rate of autoxidation of pyrogallol was recorded every 30 s for 5 min. The inhibition of pyrogallol oxidation by SOD was used to determine the enzyme’s activity.

Peroxidase (POD) activity was measured following the procedure outlined by Kim et al. (1995). A 1 mL aliquot of the supernatant was mixed with 2 mL phosphate buffer (pH 7.8), 1 mL of 0.05 M guaiacol, and 1 mL of 2% H_2_O_2_. In the blank control, 1 mL of phosphate buffer (pH 7.8) was used to replace the supernatant, while the other reagents remained unchanged. The reaction in absorbance was monitored at 470 nm. The enzyme activity was calculated based on the rate of change in absorbance.

Malondialdehyde (MDA) was measured using the thiobarbituric acid (TBA) method as described by Lei et al. (2007). Leaf and root samples (0.1 g fresh weight) were homogenized in a 10% trichloroacetic acid (TCA) solution. The mixture was then centrifuged at 9,500 rpm for 20 min at 4°C to obtain the supernatant. A 2 mL aliquot of the supernatant (for the blank control, replace the supernatant with 2 mL of 10% TCA solution) was mixed with 2 mL of 0.67% TBA solution. Subsequently, the reaction was boiled for 20 min. After cooling and centrifuging, the absorbance was measured at 450, 532, and 600 nm.

0.1 g sample of fresh plant tissue was homogenized in a 5% TCA solution. Then, the homogenate was centrifuged at 9,500 rpm for 20 min at 4°C, and the resulting supernatant was collected for the quantification of reduced glutathione (GSH) and ascorbic acid (AsA) concentrations. GSH content was quantified spectrophotometrically following the method of Ivan et al. (1988). The supernatant was mixed with 1 mL of 100 mM phosphate buffer (pH 7.7), followed by the addition of 0.5 mL of 4 mM 5,5′-dithiobis (2-nitrobenzoic acid) (DTNB) solution. For the blank control, 0.5 mL of 100 mM phosphate buffer (pH 6.8) was used in place of the DTNB solution. The reaction mixture was incubated at 25°C for 10 min, and the absorbance was measured at 412 nm.

AsA levels were determined using the method described by Islam et al. (2008). To summarize, 1 mL of the supernatant was combined with 1 mL of 5% TCA solution and 1 mL of absolute ethanol, followed by thorough mixing. Subsequently, 0.5 mL of 0.4% phosphoric acid-ethanol solution, 1 mL of phenanthroline-ethanol solution, and 0.5 mL of 0.03% FeCl_3_-ethanol solution were added. The absorbance of the reaction mixture was measured at 534 nm using a spectrophotometer. The blank control was prepared by replacing the supernatant with 1 mL of 5% TCA to account for background absorbance from reagents.

### 2.5 RNA extraction and transcriptome sequencing

The fresh root samples under the NF + Cd (no ECMF inoculation with 100 μM Cd addition), CG + Cd (CG colonization with 100 μM Cd addition), and SL + Cd (SL colonization with 100 μM Cd addition) treatments were used to extract total RNA using Trizol reagent (Invitrogen, CA, United States). The extracted RNA was quantified and purified with Bioanalyzer 2,100 and RNA 1000 Nano LabChip Kit (Agilent, CA, USA). Subsequently, cDNA library was constructed using the mRNASeq sample preparation kit (Illumina, San Diego, USA) and a paired-end RNA sequencing was performed with an Illumina HiSeq4000 at LC Sciences.

Raw sequencing reads underwent quality control processing to remove adaptor sequences, low-quality bases (Phred score < 20), and undetermined nucleotides using Cutadapt supplemented with in-house Perl scripts. Quality metrics of the cleaned reads, including Q20, Q30 scores, and GC content distribution, were systematically evaluated using FastQC.^1^ *De novo* transcriptome assembly was performed using Trinity 2.4.0 (Grabherr et al., 2011), which groups transcripts into clusters based on shared sequence content. To reduce redundancy, transcripts with sequence identity greater than 95% were grouped into the same gene cluster, and the longest transcript in each cluster was selected as the representative sequence (defined as unigene). The expression levels of unigenes were quantified using Salmon, which calculates transcript per million (TPM) values (Mortazavi et al., 2008).

Differentially expressed genes (DEGs) between treatments were identified using DESeq2 with a threshold of |log_2_ (fold change) | ≥ 1 and *p* < 0.05 (Love et al., 2014). Gene function annotation for the DEGs was performed using the Gene Ontology (GO) database, while metabolic pathways enriched with DEGs were identified through the Kyoto Encyclopedia of Genes and Genomes (KEGG) database. The GO database was developed to provide a standardized vocabulary for the definition and description of gene and protein functions across a wide range of species (Ashburner et al., 2000). And KEGG is a comprehensive database that integrates information on molecular networks, including genes, proteins, pathways, and other biological systems (Kanehisa and Goto, 2000).

### 2.6 Statistical analysis

Results are presented as means ± standard error of three replications. Significant differences between control and ECMF inoculation treatments were assessed using one-way ANOVA test with *p* < 0.05, conducted with SPSS 26.0. Heat maps were generated using an online platform (https://www.omicstudio.cn/tool), and figures were created with Origin 2021.

## 3 Results

### 3.1 Growth characteristics of willow saplings

Colonization by *C. geophilum* and *S. luteus* increased root and leaf dry weight (DW) under no Cd stress control (Supplementary Table S2). Compared to NF, leaf, stem and root DW were significantly increased by 43.84, 49.39, and 120.65%, respectively, under SL. Under Cd stress, leaf DW and shoot-to-root ratio (S:R) were notably increased by 54.55 and 125.35%, respectively, with *S. luteus* colonization compared to NF + Cd. Significant differences of root indices were observed between ECMF*-*inoculated and non-inoculated treatments (Supplementary Table S3). The root surface area, root volume, root length and average root diameter of willow saplings were significantly greater in the ECMF- inoculated treatments compared to non-inoculated treatments. Specially, root surface area and root length increased by 56.52 and 44.76%, respectively, under SL compared to NF, although these increases were suppressed under SL + Cd. In contrast, root parameters exhibited a consistently positive trend following colonization by *C. geophilum*, regardless of Cd addition.

### 3.2 Effects of ECMF on chlorophyll content and photosynthesis parameters

Compared with NF, chlorophyll contents in the NF + Cd treatment decreased by 11.06%, though this change was not significant (*p* > 0.05). In comparison to NF + Cd, chlorophyll contents significantly increased by 10.19% under CG + Cd, but decreased by 38.92% under SL + Cd (Table 1), indicating that *C. geophilum* enhanced, while *S. luteus* reduced the synthesis of photosynthetic pigments. The net CO_2_ assimilation rate (*A*) and transpiration rate (Tr) decreased by 43.10 and 25.86%, respectively, under NF + Cd, compared to NF (*p* < 0.05). Colonization of *S. luteus* had no significant effect on photosynthesis parameters except for *A*. In addition, the *A* values increased by 115.53% under CG + Cd, compared to NF + Cd, which was consistent with the changes in leaf chlorophyll contents (Table 1). The intercellular CO_2_ molar fraction (C_i_) values were reduced under all Cd treatments, including those with ECMFs colonization, consistent with the increase in *A* values (Table 1).

**Table 1:** Photosynthetic parameters of willow saplings.

Treatments	Net CO_2_ assimilation rate (μmolCO_2_ /m^2^ /s)	Stomatal conductivity (mol/m^2^ /s)	Intercellular carbon dioxide concentration (mg/kg)	Transpiration rate (mol/m^2^ /s)	Chlorophyll content (mg/g)
NF	11.09 ± 1.74b	0.50 ± 0.13a	323.66 ± 12.14a	10.44 ± 0.53b	2.08 ± 0.012b
CG	19.49 ± 0.44a	0.73 ± 0.15a	281.00 ± 19.00c	12.04 ± 1.82a	2.71 ± 0.22a
SL	13.23 ± 1.01b	0.43 ± 0.08a	299.00 ± 17.05bc	10.41 ± 1.02b	1.88 ± 0.10b
NF + Cd	6.31 ± 0.86c	0.37 ± 0.15a	343.33 ± 9.87a	7.74 ± 1.56c	1.85 ± 0.23b
CG + Cd	13.60 ± 0.51a	0.42 ± 0.005a	307.00 ± 14.74b	12.22 ± 0.27a	2.06 ± 0.12a
SL + Cd	9.32 ± 1.61b	0.39 ± 0.17a	320.33 ± 7.83a	8.06 ± 2.12bc	1.13 ± 0.069a

NF represents saplings without ECMF inoculation and Cd addition. CG and SL denote saplings that had been inoculated with *C. geophilum* and *Suillus luteus*, respectively. NF + Cd indicates saplings without ECMF inoculation but 100 μM Cd addition; CG + Cd represent saplings that had been with treated with *C. geophilum* inoculation and Cd addition (100 μM). SL + Cd represent saplings that had been with treated with *Suillus luteus* inoculation and Cd addition (100 μM).

### 3.3 Physiochemical responses of willow saplings

**Figure 1:**
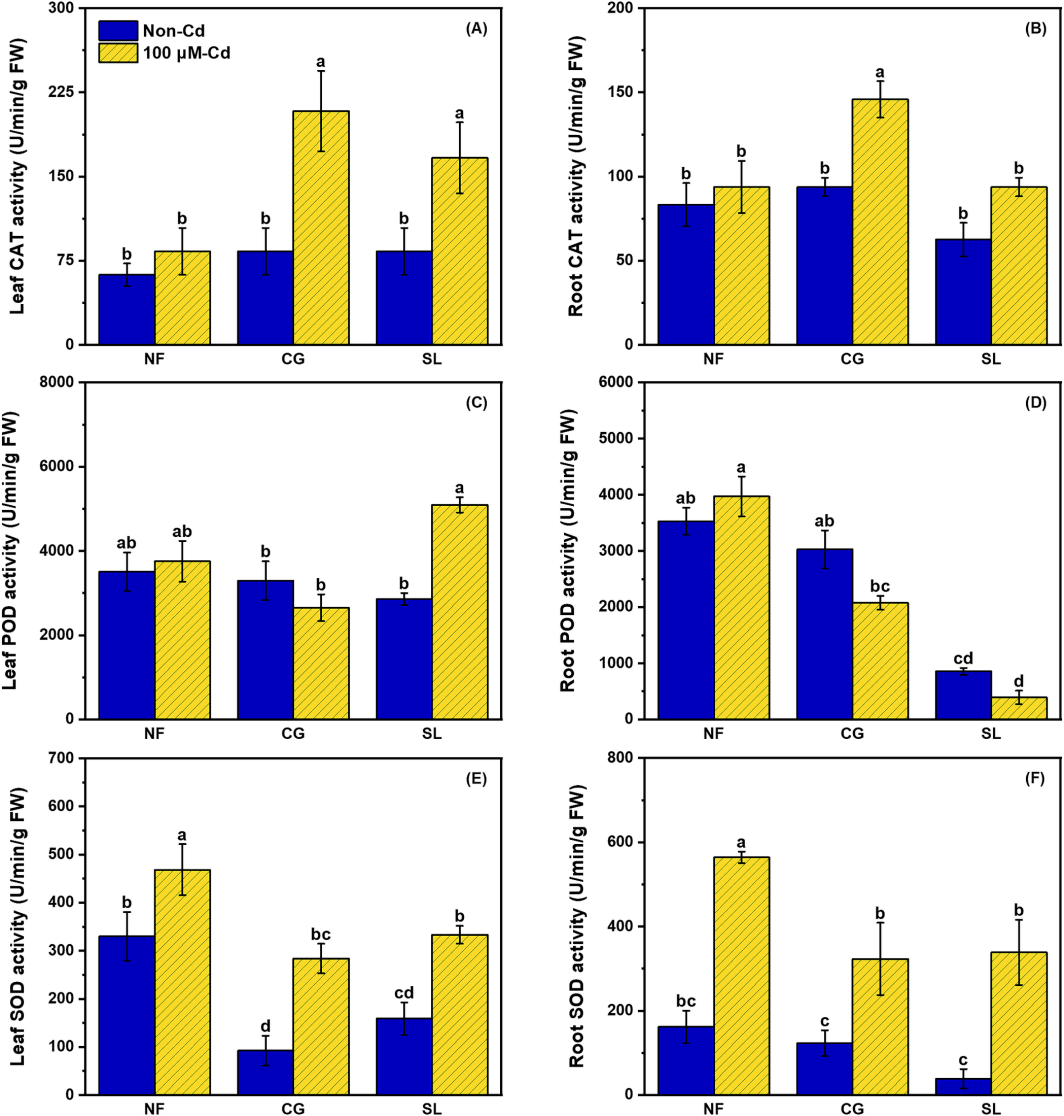
Effects of no-ECMF inoculation (NF), *Cenococcum geophilum* (CG) or *Suillus luteus* (SL) colonization and Cd addition (100 μM) on leaf and root CAT **(A and B)**, POD **(C and D)** and SOD **(E and F)** activity of willow saplings. Abbreviations: CAT, Catalase; POD, Peroxidase and SOD, Superoxide dismutase. Different letters indicate a significant difference at *p* < 0.05 by Duncan’s multiple range test.

As shown in Figures 1A,B, there were no significant differences in catalase (CAT) activity among the NF, CG and SL treatments. However, CAT activity significantly increased under Cd stress regardless of ECMF colonization. Notably, CAT activity in leaves and roots increased by 150.60% and 55.55%, respectively, under CG+Cd compared to NF+Cd (Figures 1A,B). Leaf peroxidase (POD) activity was significantly higher under SL+Cd than under CG+Cd, while the opposite was observed for root POD activity (Figures 1C,D). Most antioxidant enzyme activities increased under Cd stress, with the exception of leaf and root POD activity under CG+Cd and root POD activity under SL+Cd. Superoxide dismutase (SOD) activities in both leaves and roots were elevated in response to Cd exposure, regardless of ECMFs colonization (Figures 1E,F).

**Figure 2:**
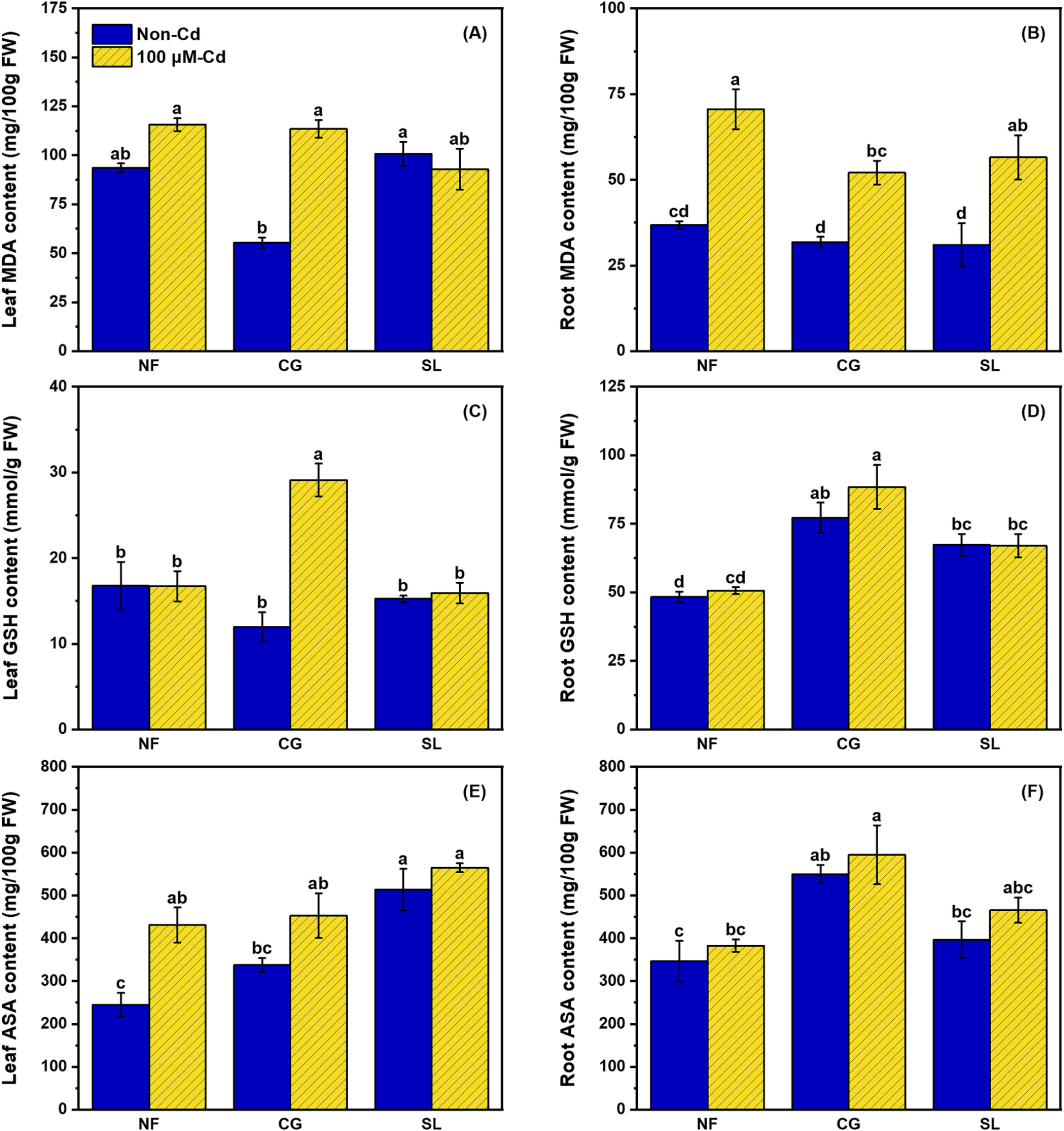
Effects of no-ECMF inoculation (NF), *Cenococcum geophilum* (CG) or *Suillus luteus* (SL) colonization and Cd addition (100 μM) on leaf and root MDA **(A and B)**, GSH **(C and D)** and ASA **(E and F)** of willow sapling. Abbreviations: MDA, Malondialdehyde; GSH, Reduced glutathione and ASA, Ascorbic acid. Different letters indicate a significant difference at *p* < 0.05 by Duncan’s multiple range test.

Malondialdehyde (MDA) content significantly increased in both leaves and roots following Cd addition (Figures 2A,B). Compared to NF, the two ECMFs notably reduced root MDA by 26.20% and 19.92% under Cd treatment (Figure 2B). Leaf and root reduced glutathione (GSH) contents exhibited a similar trend under Cd treatment. Specially, colonization of *C. geophilum* significantly enhanced leaf and root GSH content by 140.27% and 74.56%, respectively, compared to NF+Cd (Figure 2C,D). Similarly, root ascorbic acid (AsA) content was significantly higher under CG+Cd than under other treatments (Figure 2F). However, the highest AsA content in the leaves was observed under the SL+Cd treatment (Figure 2E).

### 3.4 Identification of differential expression genes under cd stress

**Figure 3:**
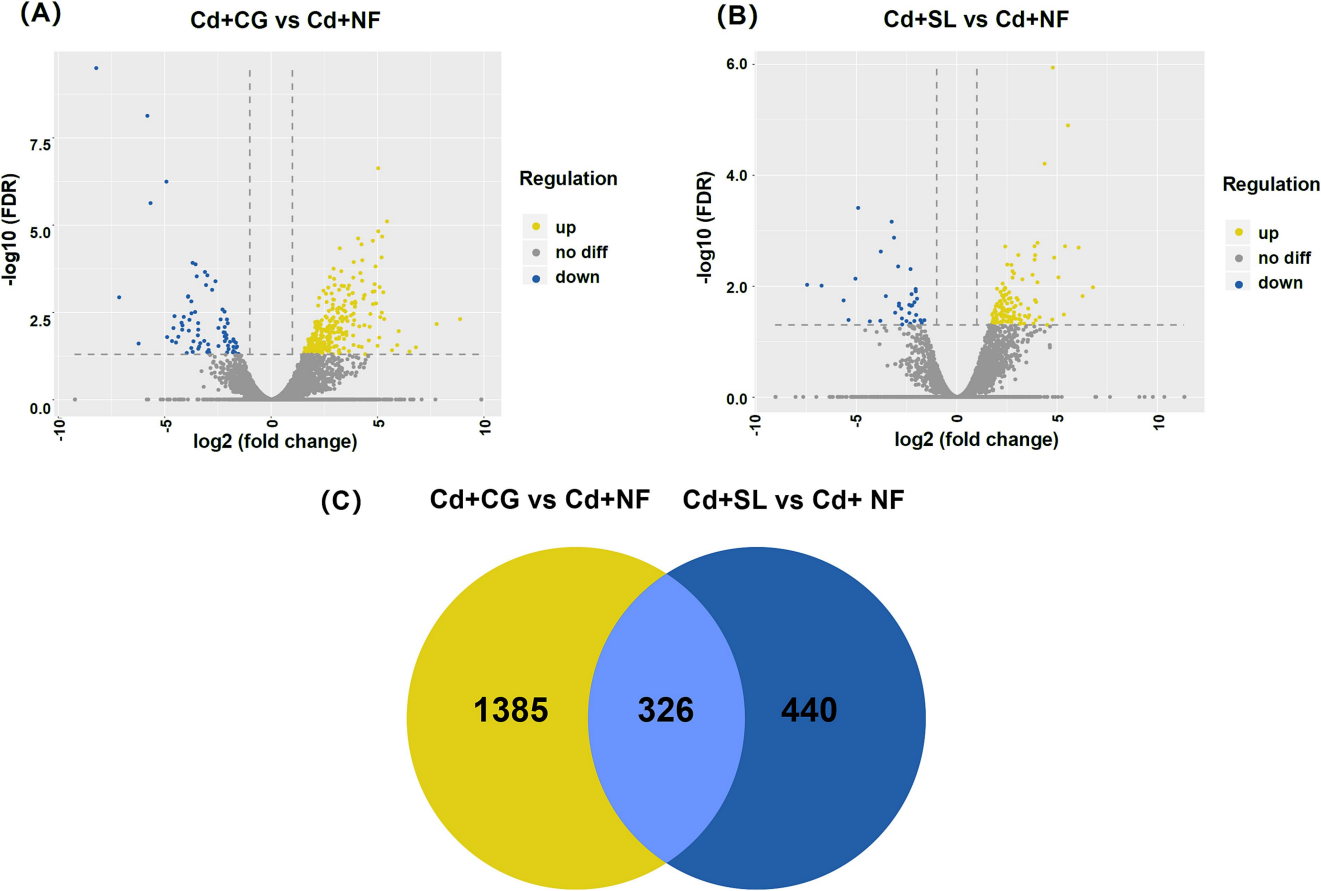
Volcano plot of different groups in **(A)** CG + Cd vs. NF + Cd and **(B)** SL + Cd vs. NF + Cd. **(C)** Venn diagram of DEGs between two groups. NF + Cd represent no ECMF inoculation (NF) with 100 μM Cd addition; CG + Cd, *Cenococcum geophilum* (CG) colonization with 100 μM Cd addition; SL + Cd, *Suillus luteus* (SL) colonization with 100 μM Cd addition.

The transcriptome analysis identified 1,711 and 766 DEGs in the CG + Cd vs. NF + Cd and SL + Cd vs. NF + Cd comparisons, respectively. This includes 1,340 and 597 upregulated genes and 371 and 169 downregulated genes, respectively. The total number of DEGs was higher in the CG + Cd vs. NF + Cd comparisons than in SL + Cd vs. NF + Cd (Figures 3A,B), indicating that the colonization with *C. geophilum* regulated more genes expression in willow saplings roots. According to The Venn diagram shows 326 DEGs common to both ECMFs colonization groups (Figure 3C). Of the DEGs, 1,385 and 440 were unique to the CG + Cd vs. NF + Cd and SL + Cd vs. NF + Cd comparisons, respectively, with 326 DEGs shared between the two comparisons (Figure 3C).

### 3.5 Functional annotation and enrichment of DEGs induced by ECMFs

**Figure 4:**
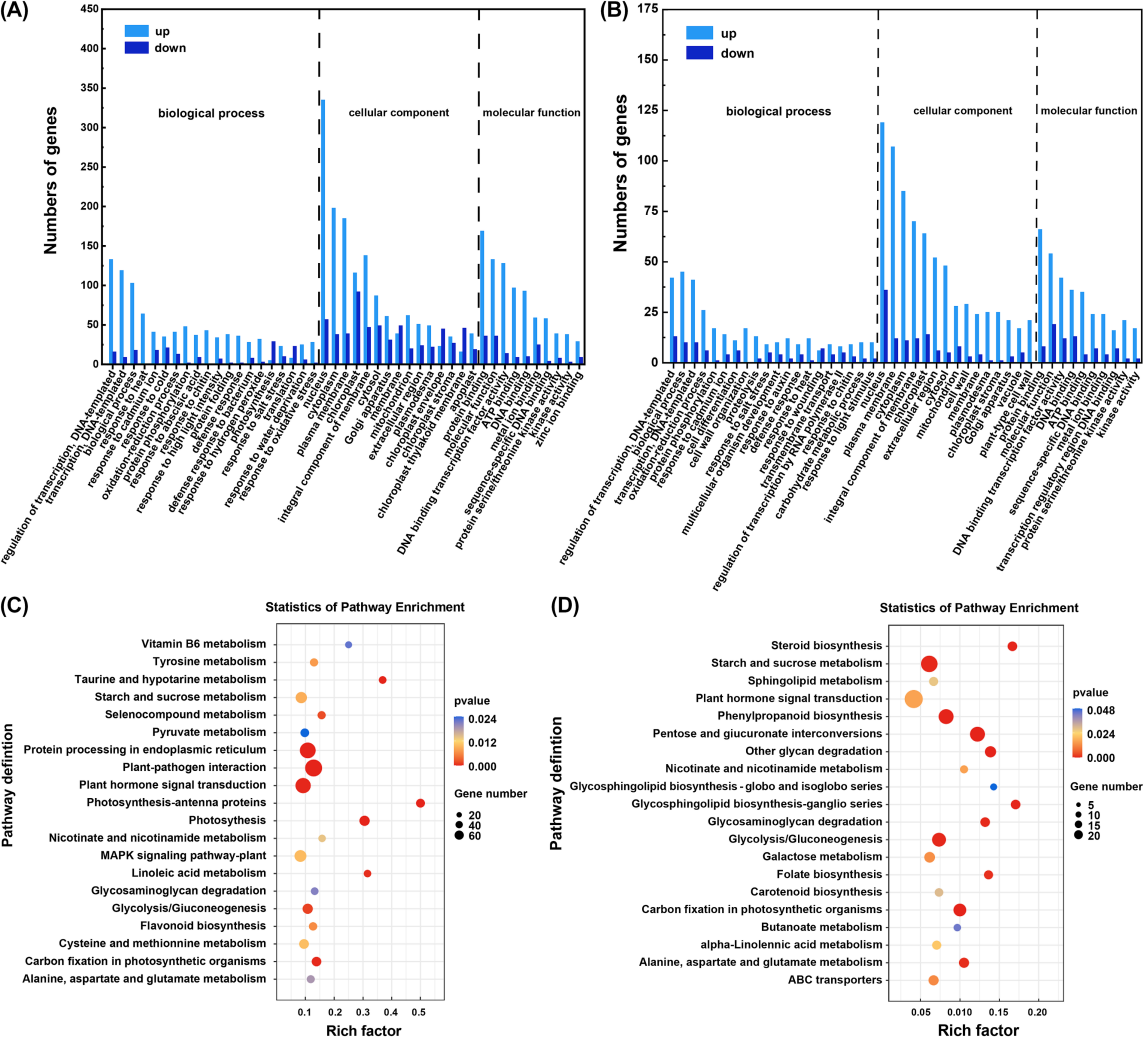
DEGs of GO and KEGG enrichment analysis. The enriched DEGs in GO analysis **(A)** CG + Cd vs. NF + Cd and **(B)** SL + Cd vs. NF + Cd. The enriched DEGs in KEGG analysis in **(C)** CG + Cd vs. NF + Cd and **(D)** SL + Cd vs. NF + Cd. NF + Cd represent no ECMF inoculation (NF) with 100 μM Cd addition; CG + Cd, *Cenococcum geophilum* (CG) colonization with 100 μM Cd addition; SL + Cd, *Suillus luteus* (SL) colonization with 100 μM Cd addition.

GO annotation classified the DEGs into three ontologies: biological process (BP), cellular component (CC), and molecular function (MF) for both pairwise comparisons. In the CG + Cd vs. NF + Cd group, 772 DEGs related to BP, 187 to CC, and 467, to MF, representing approximately 54.14, 13.11, and 32.75% of the total DEGs, respectively (Figure 4A). The DEGs associated with BP were primarily involved in ‘regulation of transcription, DNA-templated’ (GO: 0006355) and ‘biological processes’ (GO: 0008150). For CC, the main enriched terms were ‘nucleus’ (GO: 0005634) and ‘plasma membrane’ (GO: 0005886). In MF, DEGs were predominantly associated with ‘protein binding’ (GO: 0005515) and ‘molecular function’ (GO: 0003674). In the SL + Cd vs. NF + Cd comparison, 542 DEGs were related to BP, 172 to CC, and 333 to MF, accounting for 51.77, 16.43, and 31.81% of the total DEGs, respectively (Figure 4B). The distribution of DEGs in BP, CC and MF in the SL + Cd vs. NF + Cd group was similar to the observed in the CG + Cd vs. NF + Cd group.

KEGG pathway enrichment analysis was performed on the DEGs (Figures 4C,D). In the CG + Cd vs. NF + Cd group, a total of 402 DEGs were identified, with 289 genes upregulated and 113 genes downregulated in the top 20 KEGG pathways. The most significantly enriched pathways were ‘plant hormone signal transduction’ (ko04075) and the ‘mitogen activated protein kinase (MAPK) signaling pathway’ (ko04016). In contrast, the SL + Cd vs. NF + Cd group had 193 DEGs, with 168 genes upregulated and 25 genes downregulated among the top 20 KEGG pathways. Here, the most enriched pathways were ‘plant hormone signaling transduction’ (ko04075) and ‘phenylpropanoid biosynthesis’ (ko00940). Notably, the common enrichment pathways across both groups included ‘plant hormone signal transduction’ (ko04075), ‘starch and sucrose metabolism’ (ko00500), ‘glycolysis/gluconeogenesis’ (ko00010).

### 3.6 Plant hormone signaling transduction and carbohydrate metabolism pathways

**Figure 5:**
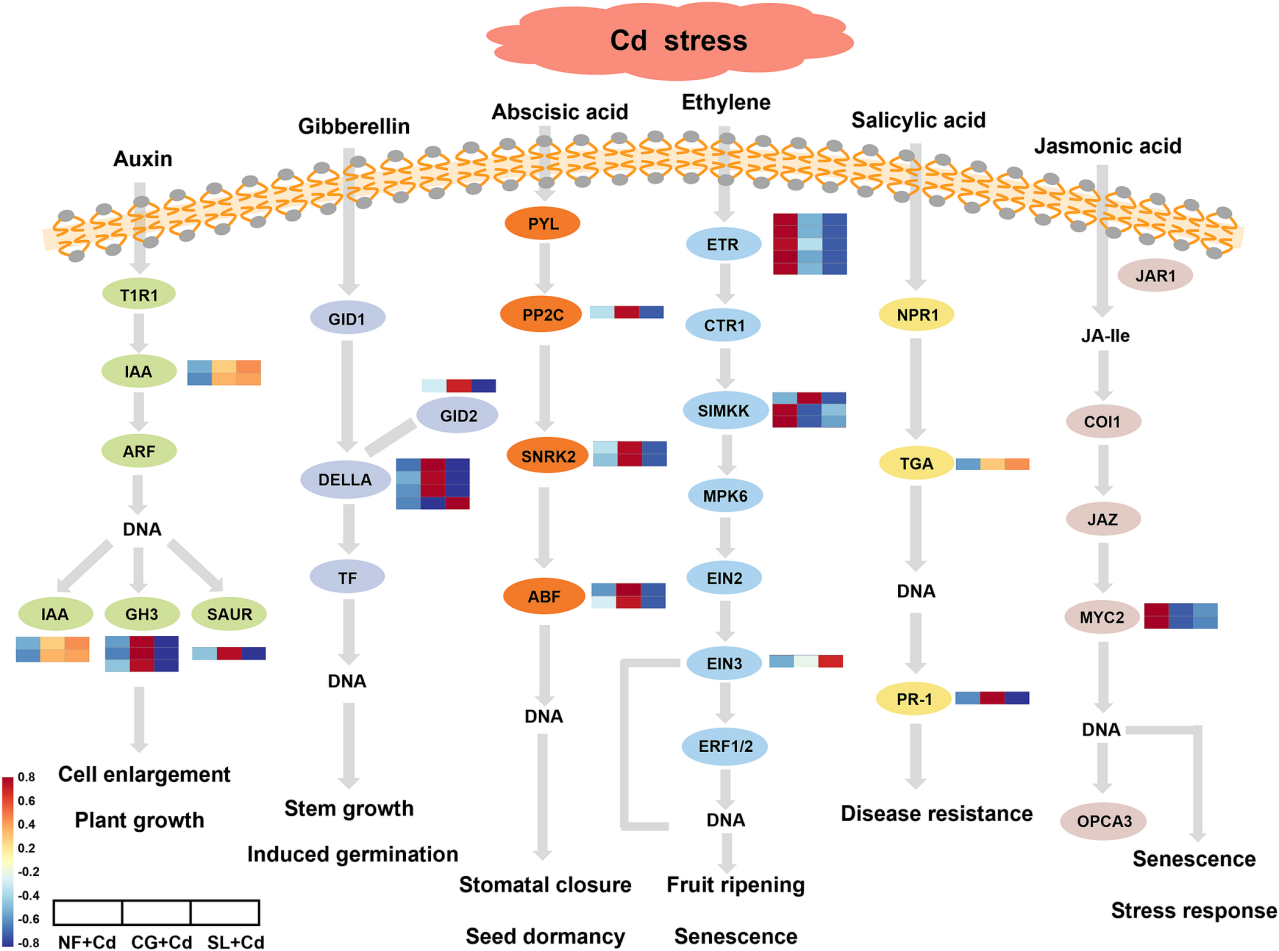
Plant hormone signaling transduction pathway in *Salix psammophila* ‘Huangpi1’ under Cd stress. NF + Cd represent no ECMF inoculation (NF) with 100 μM Cd addition; CG + Cd, *Cenococcum geophilum* (CG) colonization with 100 μM Cd addition; SL + Cd, *Suillus luteus* (SL) colonization with 100 μM Cd addition.

After colonization with ECMFs, the pathways co-enriched by both ECMFs included plant hormone signaling transduction and carbohydrate metabolism. The DEGs associated with the biosynthesis and signaling of auxin, gibberellin (GA), abscisic acid (ABA), ethylene, salicylic acid (SA) and jasmonic acid (JA) are illustrated in Figure 5. We identified six DEGs in the auxin signal transduction pathway, with IAA genes encoding indole-acetic acid proteins being significantly upregulated in ECMF-colonized saplings compared to NF + Cd. The GH3 and SAUR genes, which encode small auxin-up RNA, were upregulated under CG + Cd and downregulated under SL + Cd. Four DELLA genes, which act as negative regulators, were significantly upregulated CG + Cd compared to SL + Cd. Additionally, in the ABA biosynthesis and signaling pathway, one PP2C gene, two SnRK2 genes and two ABF genes encoding the ABA response element binding factor were upregulated under CG + Cd but downregulated under SL + Cd. In ethylene biosynthesis and signaling pathway, the ethylene receptor ETR gene and SIMKK gene encoding MAPK were downregulated in ECMF-colonized saplings, while the EIN3 genes encoding ethylene-insensitive protein were upregulated under SL + Cd and downregulated under CG + Cd. Moreover, ECMF-colonized willow saplings showed upregulation of TGA gene expression in SA biosynthesis and downregulation of MYC2 gene expression in JA biosynthesis, respectively.

**Figure 6:**
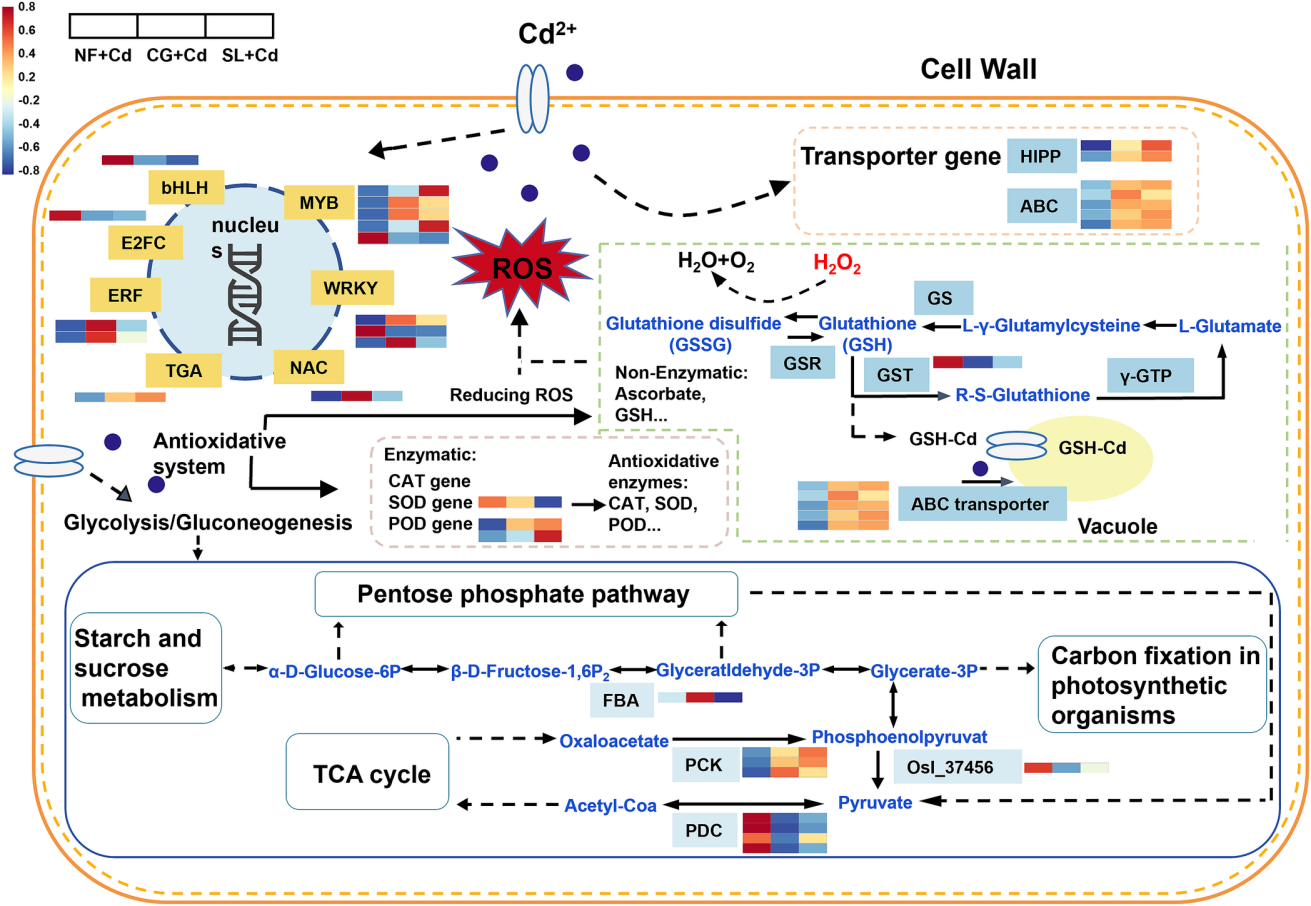
Transcriptome analysis of different treatments in *Salix psammophila* ‘Huangpi1’ under Cd stress. NF + Cd represent no ECMF inoculation (NF) with 100 μM Cd addition; CG + Cd, *Cenococcum geophilum* (CG) colonization with 100 μM Cd addition; SL + Cd, *Suillus luteus* (SL) colonization with 100 μM Cd addition.

The DEGs related to the glycolysis/glycogenesis pathway, including FBA, OsI_37456, PCK, and PDC, exhibited significant changes following ECMF colonization (Figure 6). The FBA gene, which encodes an enzyme involved in glycolysis/gluconeogenesis, was significantly upregulated under CG + Cd compared to SL + Cd. In addition, the PCK gene, involved in carbohydrate metabolism, was upregulated, whereas the OsI_37456 and PDC genes were downregulated in ECMF-colonized saplings. Notably, the changes in most glycolysis/gluconeogenesis-related genes followed a similar trend across different ECMF colonization treatments. Furthermore, compared to SL + Cd, the genes associated with starch and sucrose metabolism were more significantly upregulated under CG + Cd, which directly affected the glycolysis/gluconeogenesis pathway and TCA cycle (Supplementary Figure S1). Overall, the colonization with *C. geophilum* more effectively activated genes associated with plant hormone signaling transduction and enhanced carbohydrate metabolism, thereby improving resistance to Cd stress.

### 3.7 Heavy metal transport and transcriptional factor

Among the DEGs, seven transporter genes responsive to Cd stress were identified, including some from the ATP-binding cassette (ABC) transporter family and heavy metal-associated isoprenylated plant proteins (HIPPs) (Figure 6). Notably, DEGs encoding HIPPs were remarkably upregulated under both CG + Cd and SL + Cd. Similarly, DEGs encoding ABC transporter family, were also significantly upregulated under both treatments (Figure 6).

A total of 14 DEGs of willow saplings roots were identified as transcription factors (TFs) under Cd stress, belonging to 7 TF families (Figure 6). The largest TF family was MYB, with five DEGs (4 upregulated and 1 downregulated), followed by WRKY with three DEGs (2 upregulated and 1 downregulated), and ERF with two upregulated DEGs. MYB genes were more significantly upregulated under SL + Cd, while WRKY and ERF genes showed greater upregulation under CG + Cd. Additionally, genes from bHLH and E2FC families were downregulated, whereas genes from the TGA family were upregulated in ECMFs colonization treatments. Overall, most TF were significantly upregulated in saplings colonized by ECMFs, although some TF families, such as bHLH, were notably downregulated.

## 4 Discussion

### 4.1 ECMFs enhanced plant tolerance to cd stress

In temperate regions, ECMFs can establish ectomycorrhizal symbiosis with various dominant tress species (Rog et al., 2020). Numerous studies have demonstrated that ECMFs can alleviate the adverse effects of Cd on host plants though enhancing biomass production, nutrient uptake, modulating antioxidant defense response, and reducing the entry of HMs into plants (Yu et al., 2020; Sun et al., 2022). Cd, a toxic HMs, negatively impacts plants by disrupting essential physiological and metabolic processes, leading to biomass reduction, membrane lipid peroxidation, and impaired photosynthesis (Han et al., 2020). In this study, Cd exposure inhibited the growth of willow saplings, resulting in reduced biomass (Supplementary Table S2). It is well established that ECMF colonization enhance plant growth and alleviate the toxic effects of HM on host plants (Colpaert et al., 2011). Our findings revealed that ECMF colonization improved root growth parameter and significantly increased aboveground biomass in willow saplings, particularly with *S. luteus* under Cd stress (Supplementary Tables S2 and S3). However, the beneficial effects of ECMF colonization varied between *C. geophilum* and *S. luteus*. Specifically, *S. luteus* colonization increased root volume, whereas *C. geophilum* colonization promoted root length growth under Cd stress (Supplementary Table S3).

**Figure 7:**
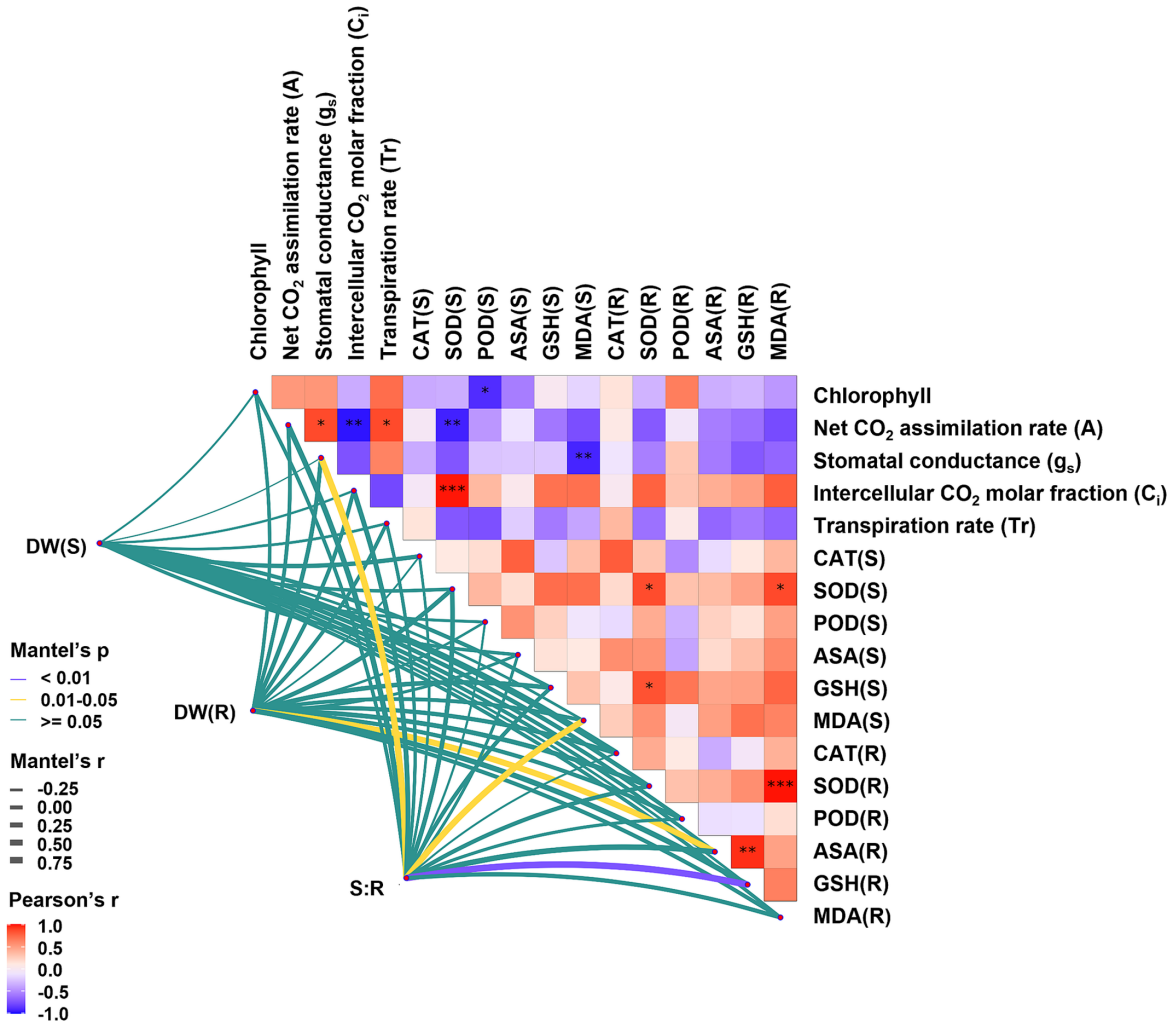
Network heatmap of growth parameters and physiological indices of *Salix psammophila* ‘Huangpi1’. DW refers dry weight of saplings. (S) meant the aerial part and (R) meant the underground part.

Photosynthesis is often adversely affected by HMs, leading to the alterations in chloroplast structure and the synthesis of photosynthetic pigments (Liu et al., 2023a). Colonization of *C. geophilum* significantly increased chlorophyll content, even in the presence of Cd stress (Table 1), suggesting that *C. geophilum* positively regulates chlorophyll synthesis (Figure 4). This enhancement likely facilitates light energy capture and supports PSII activity, thereby improving the photosynthesis rate under Cd stress (Liu et al., 2022). Typically, Cd stress reduces the photosynthesis rate in various plants, likely due to the decreased stomata opening and reduced CO_2_ assimilation rate (Xu et al., 2019). Our results indicated that ECMFs, particularly *C. geophilum*, significantly increased the net CO_2_ assimilation rate (*A*) under Cd stress (Table 1). This effect may be attributed to improved stomatal conductance (g_s_) and transpiration rate (Tr) in willow saplings, which enhance CO_2_ and water availability, thereby boosting photosynthesis and stress tolerance (Shi et al., 2019). The observed positive correlation between *A*, g_s_, and Tr supports this hypothesis (Figure 7).

Cd stress generally leads to the accumulation of reactive oxygen species (ROS) and triggers various defense responses (Li et al., 2023). Antioxidant defense systems are crucial for scavenging ROS and protecting plants against oxidative stress (Liu et al., 2023). Studies have shown that ECMF can stimulate plant antioxidant systems under Cd stress (Sun et al., 2022). In this study, ECMF colonization significantly increased catalase (CAT) activities in both leaf and root, with *C. geophilum* showing a stronger activation effect compared to *S. luteus* (Figures 1A,B). Under Cd stress, leaf peroxidase (POD) activities were significantly higher in saplings colonized by *Suillus luteus* compared to *C. geophilum*, while the opposite was observed in root POD, suggesting that more POD is synthesized in the leaves to counteract HM toxicity under *Suillus luteus* colonization (Figures 1C,D). Our results showed that a significant reduction in superoxide dismutase (SOD) activities after ECMF colonization, consist with mRNA expression level (Figures 1E,F; Figure 6). Furthermore, KEGG pathway enrichment revealed that *S. luteus* colonization was associated with phenylpropanoid biosynthesis (ko00940), whereas *C. geophilum* colonization was linked to the MAPK signaling pathway (ko04016) (Figure 4). These results suggested that *S. luteus* enhanced the synthesis of secondary metabolites, while *C. geophilum* activated signal transduction pathways involved in stress response in willow saplings. Phenylpropanoid biosynthesis, which produces lignin as an end-product in vascular plants, is identified as an important pathway under Cd stress (Yu et al., 2023a). The MAPK signaling pathway protects plants from heavy metal damage by activating transcription factors, enzymes, and proteins involved in stress response and detoxification processes (Guo et al., 2021).

Malondialdehyde (MDA) is a key marker of HM-induced oxidative stress, with excessive ROS production leading to lipid peroxidation and MDA accumulation (Liu et al., 2023). Our study demonstrated Cd stress increased MDA concentrations in both leaves and roots, indicating the oxidative damage to willow saplings, which aligns with findings from other studies (Di et al., 2024). However, colonization by both ECMFs significantly reduced root MDA content, suggesting that these fungi can mitigate Cd-induced oxidative stress and lipid damage. Besides antioxidant enzyme systems, the ascorbate-glutathione (AsA-GSH) cycle plays a crucial role in scavenging Cd-induced ROS, thereby protecting plants from oxidative stress (Wang et al., 2020). Previous study has shown that *S. luteus* colonization increased ascorbic acid (AsA) and reduced glutathione (GSH) levels in *Quercus acutissima* seedlings under Cd stress (Sun et al., 2022), and similar results were observed in in willow saplings, particularly with *C. geophilum* (Figures C–F). Furthermore, GSH can act as a precursor for the synthesis of phytochelatins (PCs) synthesis via glutathione s-transferases (GSTs), although in our study, GSTs were downregulated in ECMF colonization plant under Cd stress (Figure 6). This suggests that while GSH under ECMF colonization may not produce more PCs, it could directly chelate Cd and facilitate its sequestration in vacuolar of willow saplings (Shen et al., 2024). Moreover, there was a significant positive correlation between the S:R and MDA content in the aboveground tissues, and the similar correlation between S:R and GSH content in the underground parts (Figure 7). These results confirmed that ECMF can regulate the antioxidant enzyme and AsA-GSH cycle, thus reducing ROS production. Additionally, ROS influence various biochemical pathways, including redox signaling, phytohormone signaling, and MAPK signaling (Li et al., 2021). Similarly, Yan et al. (2022) reported that *T. harzianum* could activate defensive plant hormone signal transduction, induce genes expression involved in defense, and enhance the AsA-GSH cycle and plant antioxidant activity.

### 4.2 ECMFs regulated hormone signaling and carbohydrate metabolism under cd stress

To investigate the molecular mechanisms of saplings colonized by ECMFs in response to Cd^2+^ exposure, we analyzed the variations in the transcriptome of roots and identified 1,711 DEGs under CG + Cd treatment and 766 DEGs under SL + Cd treatment (Figure 3). This indicated more genes were activated following *C. geophilum* colonization under Cd stress. Furthermore, these DEGs were primarily enriched in the plant hormone signal transduction and carbohydrate metabolism pathways under both ECMFs treatments.

Plant hormones have the ability to induce adaptive responses, which are essential for regulating plant growth and development, as well as resisting diverse abiotic stresses (Guo et al., 2021). Our results found that the DEGs involved in plant hormone signaling transduction expressed differently under two ECMF colonization treatments in response to Cd stress. *C. geophilum* colonization upregulated IAA, GH3 and SAUR genes under Cd stress (Figure 5; Supplementary Figure S5). Similarly, Meng et al. (2022) observed that some GH3 and SAUR genes were upregulated, and the capacity of root absorption of nutrients was increased, which was conducive to plant adaptation to toxic environments. As a negative regulator, the DELLA protein has an essential influence in gibberellin (GA) signal transduction (Sun et al., 2023). Our study demonstrated that the genes encoding DELLA protein were upregulated under CG + Cd (Figure 5). Under abiotic stresses, the elevation of DELLA protein can upregulate enzyme activities that could inhibit ROS accumulation and retard programmed cell death, thus enhancing the tolerance of plants (Xin et al., 2023). In addition, previous study had shown that the abscisic acid (ABA) signaling-mediated response to Cd stress may enhance Cd tolerance by reducing Cd transport from belowground roots to aboveground tissues (Liu et al., 2023b). The PP2C, SNRK2 and ABF gene were upregulated under CG + Cd but downregulated under the SL + Cd (Figure 5). These results showed that *C. geophilum* might activate downstream 2 Snfl-related kinase (SnRK2) and ABF-binding factors (Cao et al., 2024), thereby enhancing ABA signal transduction. Besides, the accumulation of Cd content in roots was significantly increased after colonization by *C. geophilum*, reducing the transport to the aboveground part (Supplementary Figure S3). Genes encoding TGA transcription factor of in ECMF-colonized saplings were significantly upregulated (Figure 5). In addition, the MYC2 genes were downregulated in the jasmonic acid (JA) biosynthesis process under the colonization treatments (Figure 5). MYC2, a member of basic helix–loop–helix (bHLH) TF family, is a pivotal regulator in JA signaling (Li et al., 2023). Overall, our study discovered that TF under ECMF colonization could promote the biosynthesis and signaling of plant hormones, with this effect being more pronounced in *C. geophilum* colonization.

Carbohydrate and energy metabolism in willow saplings played essential roles in response to Cd stress (Figure 6). Our results found that the glycolysis/gluconeogenesis and the metabolism pathways of starch and sucrose were significantly enhanced after the colonization by ECMFs (Figure 4; Supplementary Figure S5). Carbohydrates are abundant in plants, serving as energy sources and signaling molecules to cope with abiotic stresses (Sun et al., 2023). Genes associated with starch and sucrose metabolism were highly upregulated under CG + Cd than SL + Cd, directly affecting the production of soluble sugar and TCA cycle (Supplementary Figure S1). Similarly, colonization with arbuscular mycorrhizal fungi in *P. cathayana* roots activated gene expression in the starch and sucrose synthesis pathway, enhancing the synthesis of sucrose and soluble sugar and reducing damage from abiotic stress (Han et al., 2023). Genes related to glycolysis/gluconeogenesis control energy production necessary for maintaining homeostasis under stress (Juan et al., 2021). Research on FBA (fructose-bisphosphate aldolase 2) *in vivo* suggests that this enzyme exerts metabolic control on photosynthetic CO_2_ fixation and growth (Yang and Lv, 2022), consistent with our measured photosynthetic parameters (Table 1). Under Cd stress, the PCK genes were upregulated, while the PDC genes were downregulated under EMCF-colonized treatments (Figure 6). PCKs, expressed in numerous plants, can convert lipids and certain amino acids into sugars through gluconeogenesis and regulate amino acid metabolism (Jiang et al., 2022). We speculated that ECMF colonization increases soluble sugar content in saplings, and alters energy production, and modifies ATP supply pathways. Consequently, our results suggest that the modulation of genes associated with carbohydrate metabolism under ECMF colonization, especially under *C. geophilum* colonization, enhances willow saplings tolerance to Cd stress.

### 4.3 ECMFs regulated the transporters and transcription factors under cd stress

A previous study has shown that ECMF colonization increased the Cd absorption by roots, reduces Cd translocation to aboveground parts, and decreased Cd accumulation and toxicity in plants (Zhang et al., 2023). We found that compared to non-inoculation treatments, the ECMF colonization increased Cd content in leaves, stems and roots, particularly in the roots of the CG + Cd treatment (Supplementary Figure S3). This trend, consistent with promoted enrichment factors, suggests that *C. geophilum* is more effective at accumulating Cd in roots. Transporters play a critical role in the absorption, chelation, translocation, and sequestration of HM, enhancing plant tolerance to HM stress (Jing et al., 2024). In this study, five genes involved in HM transport (including three ABCC subfamilies, one ABCF, and one ABCG subfamily member) were significantly upregulated in the roots of ECMF-colonized willow saplings (Figure 6; Supplementary Figure S5). The most expressed transporter under Cd stress in our study was ABCC transporters. It has been reported that ABC subfamily, particularly AtABCC1 and AtABCC2, can transport GSH-Cd complexes into vacuoles, playing a critical role in sequestering Cd within vacuoles (Sun et al., 2023). Thus, the upregulation of ABCC transporters in this study confirms that ECMFs colonization facilitates the transport of Cd from the cytoplasm to vacuoles, stabilizing Cd through compartmentalization. In addition, HIPPs are involved in HM detoxification in plants (Zhang et al., 2023). We found two HIPP genes (HIPP21 and HIPP36) were significantly upregulated in the roots of ECMF-colonized willow sapling under Cd stress (Supplementary Figure S5). Similarly, the expression of HIPP 20 and HIPP36 was upregulated in mulberry roots, alleviating the negative effects of Cd on vascular plants by binding to Cd (Guo et al., 2021). HIPPs can capture free Cd^2+^ in the cytosol, preventing ions from combining with essential protein and protecting plants from Cd hazards (de Abreu-Neto et al., 2013). In this study, the translocation factor was decreased under ECMF colonization treatments, indicating that ECMF could capture Cd^2+^ and fix it in plant roots (Supplementary Figure S3).

TFs can activate or repress gene expression by recognizing and binding to cis-regulatory modules, playing crucial roles in regulating stress responses (Cao et al., 2024). In this study, 14 TFs were identified and affiliated with seven families, including MYB, WRKY, ERF, NAC, TGA, bHLH and E2FC. MYB, the largest TF family, was significantly upregulated after ECMF colonization, especially under SL + Cd (Figure 7). A previous study has demonstrated that R2R3-MYBs not only participate in plant developmental processes and stress responses, but also regulated primary and secondary metabolism, particularly the biosynthesis of phenylpropanoids (Deng and Lu, 2017). In this study, the phenylpropanoid biosynthesis (ko00940) pathway was enriched under SL + Cd, consistent with the upregulation expression of MYB. WRKY TFs play an essential role in plant signal transduction feedback during abiotic stress (Yan et al., 2022). Gu et al. (2024) have proven that overexpression of ZmWRKY64 positively regulates Cd stress tolerance in *Arabidopsis* by affecting Cd absorption and transport. ERF genes also play key roles in plant tolerance to various stresses, and this family is also one of the most prominently involved in regulating primary and secondary metabolism (Zhu et al., 2023). For instance, ERF194 can regulate drought stress-related gene expression, promote antioxidant enzyme activity, and enhance the synthesis of soluble sugars, thereby alleviating the effects on poplar growth (Huan et al., 2023). In this study, both WRKY and ERF TFs were significantly upregulated after ECMF colonization, particularly under *C. geophilum* colonization (Figure 6; Supplementary Figure S5). Additionally, numerous studies have reported that TF such as NAC, bHLH and E2FC play vital roles in the tolerance mechanisms to metal stress (Liu et al., 2023b; Cao et al., 2024). Our findings support the view that different ECMFs can regulate downstream genes by modulating transcription factors to enhance resistance to HM stress.

## 5 Conclusion

This study explored the mechanism underlying the individual colonization of two ECMFs (*S. luteus* and *C. geophilum*) and their effects on modulating willow sapling Cd toxicity and tolerance, focusing on physiochemical and transcriptional insights. The colonization of ECMFs enhanced the Cd tolerance by promoting photosynthesis and modulating the antioxidant system. The effects of the two ECMFs on plant photosynthesis enzyme activities, and antioxidant substances differed, with *C. geophilum* showing superior performance. Multiple DEGs played critical roles in encoding plant hormone signal transduction, antioxidant defense system and transporters distribution in willow saplings, synergistically regulating the molecular mechanism under Cd stress. Both ECMFs commonly enriched some key KEGG functional pathways, with *C. geophilum* particularly enhancing genes associated with plant hormone signaling and carbohydrate metabolism. Overall, we revealed the molecular mechanisms of different ECMFs on Cd accumulation and tolerance. Additionally, this study confirms the potential application of ECMFs in aiding phytoremediation of Cd contaminated soil.

## Data Availability

The data presented in the study are deposited in the figshare repository, accession number: DOI: 10.6084/m9.figshare.28478033.

## Footnotes

^1^ http://www.bioinformatics.babraham.ac.uk/projects/fastqc/
