# Supplementary material for: Transcriptome analysis unveils the functional effects of ectomycorrhizal fungal colonization on cadmium tolerance of willow saplings
Source: Front Microbiol. 2025 Mar 18;16:1570200. doi: 10.3389/fmicb.2025.1570200 (PMC11958716; doi:10.3389/fmicb.2025.1570200)
Supplement: Supplementary file 1 [file Data_Sheet_1.docx]

Transcriptome analysis unveils the functional effects of ectomycorrhizal fungal colonization on cadmium tolerance of willow saplings

Text S1. Ectomycorrhizal inoculation procedure and determination of mycorrhizal colonization rate

Crush the mycelium of two ECMF cultured in liquid culture using a blender as inoculant, and mix the sterilized water and inoculant in a ratio of 3:1 to prepare the inoculant solution before inoculation. Use a small shovel to gently turn over the soil, and inoculate 50 mL of inoculum into the corresponding treatments respectively, so that the root of willow saplings can fully contact with the solution. In the control group, use the same amount of sterile water for irrigation, and finally cover a small amount of soil on the surface of the potted plants. The inoculation will be conducted once every 2 weeks for a continuous period of 3 months.

During the inoculation period, the mycorrhizal colonization rate of ECMF should be observed and measured every month. Root samples of willow saplings should be randomly collected, washed with distilled water, and cut into 1cm long root segments. The mycorrhizal colonization rate (the proportion of mycorrhizal root segments to all root segments) should be observed under a microscope using staining method. If it reaches 60% or more, the next step of Cd stress test can be carried out.

Text S2. RNA-seq validation of the accuracy of transcriptome

Quantitative real-time PCR (qRT-PCR) was performed to verify the accuracy of RNA-seq data. PCR reaction conditions were as follows: 1 cycle at 95℃ for 30 seconds, 40 cycles at 95℃ for 5 seconds, and 40 cycles at 60℃. Gene expression levels were calculated using the 2^−∆∆Ct^ method. The gene primer sequences used are listed in Table S1. The *q*RT-PCR results were consistent with RNA-seq, which was listed in Figure. S2. The results showed similar trends in gene expression changes, although the extracted expression levels varied slightly. This confirmed the reliability of the transcriptome sequencing results.

Table S1. Primers used in qRT-PCR

| Gene | primer F（5-3'） | primer R（5-3'） |
| --- | --- | --- |
| SDH | CAGTCTGTGCTCAAAGAGGATATT | CTTGGGGTCCAAGAGAAGGG |
| PSBR | GGGAGCCCTTCTTGTTTACACT | TAGAACAGTTTTTCGGAACTGGG |
| S6PDH | ACTTGGAGCATCGGTAGCAG | CTTCCATCCGCCATACACCT |
| RPM1 | CACAGTGCGAAACGTGCAG | TCGTAGGGGTGACTTCTCCA |
| PER42 | CCGACAAGAGGACTAAGCCG | GCTTGGGTTTGACATGCGG |
| HSP70 | ACCAAACCGTCACTAGATCG | ACCACCATCCGAATTGACAGG |
| CAD | AAACAGCAAGCCACCCACC | AATTGAACGGGGAGAGGACC |
| MPK3 | GCTGAGCTTTTCCCACTTGTT | GCTTTCATGACCTTTAGCAACATA |
| PCK1 | TCGAACTCGAGCGTGTTATGT | CATGATTGAGGGGCGTGCTA |
| GGAT2 | TGGTGCCAGCAAAGGTGTTA | ACTGTGGCACTGGAACCAAA |

Table S2 Dry weight and S: R of different treatments

| Treatments | | Dry weight (g) | | | S: R |
| --- | --- | --- | --- | --- | --- |
|  |  | Leaf | Stem | Root |  |
| NF | 0.73±0.23b | 2.45±0.63ab | 0.92±0.31b | 3.64±0.35ab |  |
| CG | 1.28±0.49a | 1.98±0.34b | 0.83±0.14b | 3.84±0.51ab |  |
| SL | 1.05±0.46a | 3.66±0.43a | 2.03±0.83a | 2.72±0.53ab |  |
| NF+Cd | 0.66±0.45bc | 2.38±0.13a | 1.37±0.25a | 2.17±0.11b |  |
| CG+Cd | 0.57±0.025c | 1.91±0.23b | 0.99±0.21a | 2.62±0.26ab |  |
| SL+Cd | 1.02±0.11a | 2.28±0.56ab | 1.17±0.76a | 4.89±1.64a |  |

Note: NF represents saplings without ECMF inoculation and Cd addition. CG and SL denote saplings that had been inoculated with *C. geophilum* and *Suillus luteus*, respectively. NF+Cd indicates saplings without ECMF inoculation but 100 μM Cd addition; CG+Cd represent saplings that had been with treated with *C. geophilum* inoculation and Cd addition (100 μM). SL+Cd represent saplings that had been with treated with *Suillus luteus* inoculation and Cd addition (100 μM).

Table S3. Root parameters of willow saplings

| Treatments | Root surface area  (cm^2^) | Root volume  (cm^3^) | Root length  (cm) | Average diameter  (mm) |
| --- | --- | --- | --- | --- |
| NF | 368.15±40.12b | 3.94±0.82c | 2797.81±297.02b | 0.83±0.14b |
| CG | 504.46±66.13a | 5.59±.02bc | 3700.04±308.24a | 1.32±0.025a |
| SL | 576.26±67.34a | 6.55±1.33a | 4049.37±402.17a | 1.37±0.04a |
| NF+Cd | 306.01±39.02b | 2.59±0.32b | 2883.55±294.11b | 0.67±0.003b |
| CG+Cd | 412.28±28.09a | 4.10±0.56a | 3305.04±254.32a | 0.80±0.034a |
| SL+Cd | 407.92±36.91a | 4.65±0.67a | 2924.04±241.03b | 0.90±0.02a |

Note: NF represents saplings without ECMF inoculation and Cd addition. CG and SL denote saplings that had been inoculated with *C. geophilum* and *Suillus luteus*, respectively. NF+Cd indicates saplings without ECMF inoculation but 100 μM Cd addition; CG+Cd represent saplings that had been with treated with *C. geophilum* inoculation and Cd addition (100 μM). SL+Cd represent saplings that had been with treated with *Suillus luteus* inoculation and Cd addition (100 μM).

Figure S1. Starch and sucrose metabolism pathway in (A) CG+Cd vs NF+Cd and (B) SL+Cd vs NF+Cd group. Note: NF+Cd indicates saplings without ECMF inoculation but 100 μM Cd addition; CG+Cd represent saplings that had been with treated with *C. geophilum* inoculation and Cd addition (100 μM). SL+Cd represent saplings that had been with treated with *Suillus luteus* inoculation and Cd addition (100 μM).

Figure S2. qRT-PCR was used to validate the unigene of RNA-seq data. Note: NF+Cd indicates saplings without ECMF inoculation but 100 μM Cd addition; CG+Cd represent saplings that had been with treated with *C. geophilum* inoculation and Cd addition (100 μM). SL+Cd represent saplings that had been with treated with *Suillus luteus* inoculation and Cd addition (100 μM).

Figure S3. Effects of no-ECMF inoculation (NF), *Cenococcum geophilum* (CG) or *Suillus luteus* (SL) colonization of (A) Cd content on willow leaf, stems, and roots. (B) translocation factor and enrichment factor of willow saplings.

Figure S4. Heat map of relative expression levels of predicted DEGs involved in plant hormone and signal transduction. Note: NF+Cd indicates saplings without ECMF inoculation but 100 μM Cd addition; CG+Cd represent saplings that had been with treated with *C. geophilum* inoculation and Cd addition (100 μM). SL+Cd represent saplings that had been with treated with *Suillus luteus* inoculation and Cd addition (100 μM).

Figure S5. Heat map of relative expression levels of predicted DEGs involved in transcriptional factor, heavy metal related, antioxidant related and glycolysis/glycogenesis. Note: NF+Cd indicates saplings without ECMF inoculation but 100 μM Cd addition; CG+Cd represent saplings that had been with treated with *C. geophilum* inoculation and Cd addition (100 μM). SL+Cd represent saplings that had been with treated with *Suillus luteus* inoculation and Cd addition (100 μM).
